# Practical Notes and Formulæ

**Published:** 1880-01-20

**Authors:** 


### PRACTICAL NOTES AND FORMULÆ.

**Calomel—Its Conversion into Corrosive Sublimate.**—Dr. G. Vulpius (*Archiv. Pharmacia—Boston Medical Journal*, August 28, 1879). has made a considerable number of researches to determine under what conditions, if any, calomel is converted into corrosive sublimate in ordinary prescriptions.

He concludes: 1. No corrosive sublimate forms in twenty-four hours in mixtures of calomel with white sugar, milk sugar, calcined magnesia, carbonate of magnesium, or bicarbonate of sodium. 2. No such formation takes place in three months in mixtures of calomel with calcined magnesia, carbonate of magnesium and sugar. 3. Mere traces of corrosive sublimate are found at the end of three months in a mixture of calomel, bicarbonate of sodium and milk sugar. 4. A considerable quantity of corrosive sublimate forms in the same time in a mixture of calomel, sodium bicarbonate and cane sugar. 5. Corrosive sublimate forms in calomel powders containing calcined magnesia or sodium bicarbonate, if digested with water. 6. The formations of corrosive sublimate in mixtures of calomel and alkalies digested in water for a short time is not favored by the presence of hydrochloric acid, but, on the contrary, hindered to some extent, on account of the neutralization of the alkalies by the acid.—*Detroit Lancet.*

**Cold Cream without Oil.**—A new cosmetic is now in vogue in place of the old-fashioned cold cream, which it much resembles in appearance, with the advantages of being less expensive and less liable to rancidity. Mr. E. Lebaigne gives for it the following formula:

Quince seed mucilage....10 drachms.

Almond oil soap..............15 grains.

Stearic acid................ 2½ drachms.

Glycerine.................. ½ drachm.

Rub the stearic acid and the soap together in a warm mortar, add gradually to the mixture the mucilage so as to form an emulsion, and lastly the glycerine. The cream may now be perfumed with otto of rose or any suitable essential oil.—*Druggists' Circular.*

**Treatment of Prostatic Enlargement.**—Ergot internally is strongly advocated in this complaint by Mr. R. Harrison, in the *Lancet.* He sums up all treatment by saying that the great point to aim at is to secure healthy urine, and this is attained by tying in an elastic catheter, and allowing the urine to pass away as it is secreted, until it becomes healthy, and then draw it off at regular and gradually prolonged intervals.

**Tasteless Saline Purgatives.**—In the *Paris Medical*, August, 1879, Dr. Yoon recommends the following combination as almost concealing the disagreeable taste of Epsom salts:

℞. Magnesii sulphatis.................. ʒ v

Essentiæ menthæ......................gtt.iij. M.

To be dispensed in a very little water.

**Cure of Rheumatism.**—Mrs. E. A., aged twenty-seven years; first visit March 11th, 1877; pulse 100, temperature 103½, tongue very much coated, urine scanty, bowels constipated, wrist, shoulder and ankle of left side swollen, red, sensitive to touch or motion, and very painful. I prescribed as follows:

℞. Bicarbonate of potassa............℥ j

Bicarbonate of soda...............ʒ vj. M.

Divide into 15 parts and put up in blue papers.

Citric acid...........................℥ ss.

Divide into 15 parts and put up in white papers.

SIG.—Dissolve one of each kind in one-third tumbler of water, mix and drink while effervescing.

℞. Fl. ext. aconite.......................gtt.xx
Water..............................℥ iv. M.

SIG.—Teaspoonful every hour.

March 13.—Patient quite easy; continued effervescing powders; discontinued aconite; prescribed two grains of quinine every four hours; alternating with effervescent powders. March 16.—Patient convalescent; continued effervescent powders once in six hours, alternating with quinine. March 20.—Patient quite well, and at work.

I have followed this treatment in quite a number of cases, and with good results. I sometimes have to treat complications, but place my reliance on the potassa and soda as general remedies in this disease.—*Michigan Medical News.*

**Whooping Cough Mixture.**—In pertussis, Dr. Pollak, of Austria, recommends, for insufflation:

℞. Quiniæ tannatis.......................
Sodii bicarbonatis...................aa 5 parts
Pulv. acaciæ........................ 100 parts.

Use with an insufflator.

—*Medical and Surgical Reporter.*

**Ovarian Dysmenorrhœa.**—The following combination is well spoken of in the *Medical Brief*, by Dr. Pattee, in the above disease:

℞. Tinct. pulsatillæ.........................
Tinct. acteæ albæ.........................
Tinct. cimicifugæ...................aa gtt.xv
Aquæ..............................f. ℥ iv.

A teaspoonful three or four times a day.

**Quinia and Iron Tonic**—

℞. Ferri et quiniæ citr.............grains 40

Acidi hydrobrom..............fl. ʒ 2

Spirit. limon.......................fl. ʒ 1

Syrupi..............................fl. ʒ 6

Aquæ...... .......... q. s. ad fl. ℥ 6

Dose: ½ to 1 fl. ℥.—*Pharm. Journal.*

**For Syphilis.—**

*Mist. Hydrarg. Bichlor.*

℞ Hydrarg. chlor. corros.................................... gr. 1.

Potass. iodidi............................................ ʒ 2.

Tinct. gent. co........................................fl ℥ 4.

Dose, a teaspoonful.

*Mist. Potass. Iodidi.*

℞ Potass. iodidi............................................ ʒ 4.

Syr. sarsap. co............................................

Tinct. gent. co......................................aa fl ℥ 1. M.

Dose, a teaspoonful.

*Mixed Treatment: Taylor's.*

℞ Hydrarg. biniodidi........................................ gr. 1.

Potass. iodidi............................................ ʒ 4.

Syr. sarsap. co............................................

Aqua...................................................aa ℥ 2. M.

Dose, a teaspoonful.

*Mixed Treatment: Thompson's.*

℞ Hydrarg. biniodidi........................................ gr. 1.

Potassii iodidi............................................ ʒ 3.

Tinct. aurantii........................................fl ℥ 1.

Aqua...................................................fl ℥ 3. M.

Dose, a teaspoonful.

**Discutient Ointment.—**

℞ Extract of conium........................................ ʒj.

Iodide of potash.......................................... ʒj.

Extract of belladonna..................................... ʒj.

Extract of hyoscyamus..................................... ʒj.

Axungia, or glycerine of starch........................... ℥j. M.

The above will exercise a manifest resolvent action upon glandular enlargements. In similar cases, and when it is necessary to leave the extract of conium in constant contact with the diseased parts, in strumous adenitis, and chronic arthritis of the same nature, it will be more convenient to employ the emplastrum conii, the formulæ for which are numerous, but which may be made simply by spreading on a piece of skin, a plastic mass, composed of one part of white wax, two parts of resin and nine parts of alcoholic extract of conium.—*Le Progres Med.*

**Asthma.—**

℞ Iodide of potassium...................................... ʒij.

Decoction of senega....................................... ʒiij.

Tinct. of lobelia......................................... ʒvj.

Camphorated tinct of opium................................ ʒvj. M.

One to two teaspoonfuls three to four times per day.

**Treatment of Asthma.—**

℞ Senega root.......................................... ʒ ss.

Boil in

Water.......................................... ℥ jv, until the

Decoction be reduced to.......................................... ℥ ij, filter and add of

Iodide of potassium.......................................... ʒ ij.

Syrup of opium.......................................... ℥ iijss.

Brandy .......................................... ℥ ij.

color with

Tinct. of coch.......................................... q. s.

and filter.

The patient should every day take three tablespoonfuls of this elixir, one in the morning before breakfast, at mid-day and in the evening until the cessation of the asthma. This is almost, as Trousseau remarked, forty-five grains of iodide of potash per day.—*Le Progres Medical.*

**To Arrest Vomiting During Pregnancy.—**

℞ Ceri oxalat..........................................

Ipecacuanhae..........................................aa gr. i.

Creosoti.......................................... gtt. j.

M. Sig. To be taken every hour.—*New Remedies.*

**Cough Mixture.—**

℞ Cod-liver oil.......................................... ℥ ii.

Honey.......................................... ℥ ii.

Lemon juice.......................................... ℥ ii.

One to two teaspoonfuls three times a day.

**Linimentum Potassii Iodidi—**

℞. Saponis albi.......................... ʒ 12

Aquae destillatae..........................fl. ℥ 3½

Alcoholis..........................fl. ℥ 1

Potassii iodidi.......................... ʒ 6

Adipis.......................... ℥ 1

Macerate the soap in 2 ℥ of the water; dissolve the iodide of potassium in the remainder of the water; then add the alcohol, and afterwards the lard, using a gentle heat. If desired, add some perfume.—*Pharm. Journ.*

**Powder for Flatulent Dyspepsia.—**

℞ Powdered nux vomica.......................................... 10 grains.

Powdered rhubarb.......................................... 60 grains.

Prepared chalk.......................................... 45 grains.

Oleo saccharate of mint.......................................... ʒ j.

Mix carefully and divide into 20 powders. One powder before each meal in a wafer. Two tablespoonfuls of lime water in half a tumblerful of sweetened water after each meal. Wine diluted with orezza-water if the dyspepsia be complicated with anemia.

